# Bibliometric mapping of the 100 most-cited publications on optic nerve and optic pathway glioma: A cross-sectional bibliometric analysis

**DOI:** 10.1097/MD.0000000000050950

**Published:** 2026-10-02

**Authors:** Muhammad Hamza Shah, Aditya Gaur, Pearl Ohenewaa Tenkorang, Fionnuala McKeown, Subham Roy, Sakshi Roy, Umar Akram, Khabab Abbasher Hussien Mohamed Ahmed

**Affiliations:** a School of Medicine, Queen’s University Belfast, Belfast, United Kingdom; b Centre for Anatomy, Edinburgh Medical School, Edinburgh, United Kingdom; c Yeovil District Hospital, Somerset NHS Foundation Trust, Yeovil, United Kingdom; d School of Medicine, University of Ghana, Accra, Ghana; e Hull York Medical School, University of York, York, United Kingdom; f Department of Medicine, Allama Iqbal Medical College, Lahore, Pakistan; g Faculty of Medicine, University of Khartoum, Khartoum, Sudan; h Department of Pathophysiology, School of Medicine, St. George’s University, St. George’s, Grenada, West Indies.

**Keywords:** bibliometric analysis, neurofibromatosis type 1 (NF1), optic pathway glioma (OPG), pediatric neuro-oncology

## Abstract

**Background:**

Optic pathway gliomas (OPGs), including optic nerve gliomas, are uncommon tumors of the anterior visual pathway that arise mainly in young children, often with neurofibromatosis type 1 (NF1). The literature is now substantial, but its structure is undescribed. We mapped the most-cited OPG publications to characterize citation patterns, contributors, collaboration, and thematic development.

**Methods:**

Scopus was searched on April 30, 2025 using a title–abstract–keyword strategy combining 4 OPG-specific phrases, with no year or document-type restriction. Records were ranked by total citations, and the 100 most-cited were retained. Two reviewers screened independently with a 3rd adjudicating; each was then classified by how directly it addressed OPG. Metadata were analyzed in Bibliometrix and VOSviewer. Distributions were assessed with the Shapiro–Wilk test and summarized as medians with interquartile ranges (IQRs); associations used Spearman correlation. Reporting follows the preliminary guideline for reporting bibliometric reviews of the biomedical literature.

**Results:**

The 100 records, published between 2000 and 2021, were cited 9305 times, with a median of 75 citations per article (IQR 59–108; range 41–289) and a median annual rate of 5.7 (IQR 4.2–7.8). Output peaked in 2010 (n = 9) and again in 2012, 2015, and 2016 (n = 8 each). First authors spanned 17 countries, 53 from the United States. Washington University School of Medicine appeared most often, and David H. Gutmann and Robert A. Avery were the most central authors. Neuro-Oncology (n = 9), Cancer Research (n = 5), and the International Journal of Radiation Oncology Biology Physics (n = 5) led; about four-fifths appeared in 1st-quartile journals. Keyword co-occurrence formed 3 clusters: clinical/chemotherapy, NF1 molecular biology, and imaging/diagnostics. Recent articles were cited faster than older ones (rho = 0.475, publication year vs. annual rate; *P* < .001). Excluding the 9 peripherally related records left every statistic essentially unchanged (median 76; IQR 59–109).

**Conclusion:**

Among the most-cited OPG publications, emphasis has shifted over 2 decades from descriptive clinical series toward NF1 tumor biology, mitogen-activated protein kinase kinase-directed therapy, and quantitative imaging, with output concentrated in a few North American centers. These findings describe how attention has been distributed, not clinical importance or study quality, and are subject to the known limits of citation analysis.

## 1. Introduction

Optic pathway gliomas (OPGs), a designation that encompasses optic nerve gliomas, are benign tumors of the precortical visual pathway that affect mainly children and adolescents.^[1,2]^ They account for 2% to 5% of childhood central nervous system tumors and are an important consideration in the assessment of visual loss in young patients.^[3]^ OPGs may occur sporadically or in association with neurofibromatosis type 1 (NF1), an inherited tumor predisposition syndrome.^[1,2]^ Roughly 1 in 5 children with NF1 will develop an OPG.^[4]^ The tumors may involve any part of the visual pathway, including the optic nerve, chiasm, tracts, and radiations, as well as the hypothalamus.^[1,5]^

Diagnosis usually rests on neuroimaging interpreted alongside careful clinical assessment.^[6]^ Sporadic tumors typically involve a single optic nerve, whereas NF1-associated disease may be unilateral or bilateral.^[6]^ Biopsy is not generally required and carries a risk of visual loss.^[7]^ Where tissue is obtained, the predominant histological subtype is World Health Organization grade I pilocytic astrocytoma, although pilomyxoid astrocytoma and grade II diffuse fibrillary astrocytoma have also been reported.^[8]^

Management is complicated by the unpredictable behavior of these tumors.^[6]^ Some remain radiologically stable for years, while others enlarge either slowly or rapidly.^[6]^ Growth by itself does not necessarily justify intervention, since treatment decisions turn principally on visual status.^[9]^ Therapy is usually reserved for children with progressive visual loss or for those with established loss who are at high risk of further deterioration. Because “progression” has no agreed definition, treatment thresholds differ between centers. Reduced visual acuity and change on neuroimaging are the commonest triggers for intervention, with a loss of 0.2 logMAR (minimum angle of resolution) or more generally regarded as clinically meaningful.^[10]^ Visual field loss is also accepted as evidence of progression, although formal perimetry is difficult to obtain reliably in the young children in whom these tumors most often present.^[11]^

The literature on OPG is now considerable and has been approached from several directions. However, no bibliometric evaluation has yet examined how that literature is structured – which countries, institutions, and investigators have contributed most, how collaboration is organized, and how the thematic emphasis of highly cited work has shifted over time. Bibliometric analysis offers a systematic way of describing scientific output and the relationships between the groups that produce it.^[12,13]^ It can also identify recurring topics and the points at which a field’s attention has moved, which is useful when planning future work.^[14,15]^ On that basis, we undertook a bibliometric analysis of the most-cited OPG literature. Our aim was descriptive: to characterize publication and citation patterns and the thematic composition of the field, rather than to evaluate the quality or clinical value of individual studies.

## 2. Materials and methods

### 2.1. Study design and reporting standards

This was a cross-sectional bibliometric analysis of published biomedical literature. Reporting follows the preliminary guideline for reporting bibliometric reviews of the biomedical literature, which specifies minimum requirements for describing the search, the selection process, the analytic tools, and the presentation of results.^[16]^ No protocol was registered in advance, since bibliometric analyses are not eligible for registration in International Prospective Register of Systematic Reviews.

### 2.2. Ethical considerations

The study used only published, publicly indexed bibliographic records. It involved no human or animal participants, no identifiable personal information, no biological material, and no clinical intervention. Institutional review board or ethics committee approval was therefore not required, and the requirement for informed consent did not apply. This position is consistent with the exemption criteria applied to research using publicly available, non-identifiable data.

### 2.3. Data source

Scopus (Elsevier B.V.) was searched on April 30, 2025. Scopus was chosen because it provides standardized metadata for affiliations and author identifiers, broad coverage of biomedical journals, and citation counts that can be exported in a form suitable for Bibliometrix and VOSviewer.^[17]^ All records were retrieved on a single date so that citation counts, which change continuously, refer to a fixed point in time. The consequences of relying on 1 database are addressed in the Limitations.

### 2.4. Search strategy

The following query was executed in a single search, with no restriction on publication year:

TITLE-ABS-KEY (“optic pathway glioma” OR “optic nerve glioma” OR “optic glioma” OR “visual pathway glioma”) AND (LIMIT-TO (DOCTYPE, “ar”) OR LIMIT-TO (DOCTYPE, “re”)) AND (LIMIT-TO (LANGUAGE, “English”))

The 4 phrases were chosen because they are the terms under which this tumor is indexed and reported in the ophthalmic, neurosurgical, and neuro-oncological literature, and because each is specific: none of them retrieves general glioma literature when searched as a bound phrase. Truncation was deliberately avoided so that the phrases would not broaden into unrelated compounds. Document types were limited to articles and reviews, in keeping with the exclusion of editorials, letters, and conference material, and the language limit reflects the practical constraint that the review team could appraise relevance only in English.

Two features of the Scopus title–abstract–keyword field are relevant to the interpretation of what was retrieved. First, the field searches author keywords and Scopus index keywords in addition to the title and abstract, so a record can be returned when 1 of the 4 phrases appears only as an indexed descriptor. Second, index keywords are assigned to describe the anatomical structures and disease entities discussed anywhere in a paper. A surgical or imaging study in which the optic pathway is discussed as adjacent anatomy, or an NF1 study in which optic glioma is listed among the manifestations of the syndrome, can therefore be retrieved even though the tumor is not the subject of the work. This is the mechanism by which a small number of peripherally related records entered the citation-ranked set, and it is the reason a formal relevance assessment was added at the eligibility stage (Section 2.5).

Because a single database was searched, no cross-database duplicates were generated, and no deduplication step was required. Scopus was checked for within-database duplicate records; none were identified.

### 2.5. Eligibility criteria and relevance assessment

Records were eligible if they were original research articles, review articles, or case series; were published in English; and addressed optic pathway or optic nerve glioma in a clinical, epidemiological, diagnostic, therapeutic, or laboratory context. Editorials, commentaries, letters to the editor, conference abstracts, errata, and book chapters were excluded, as were records concerned solely with other central nervous system tumors or with unrelated ocular disease.

A citation-ranked set assembled through index-term retrieval will not be uniformly on topic, and we therefore added an explicit relevance assessment. Two reviewers (MHS and SaR) independently examined the full record – title, abstract, keywords, and, where necessary, the full text – of each of the 100 articles and assigned it to 1 of 3 categories. Disagreements were resolved by discussion with a 3rd reviewer (SuR). Throughout this article, SaR denotes Sakshi Roy, and SuR denotes Subham Roy. The categories were defined in advance as follows:

• Category A: Optic pathway or optic nerve glioma is the primary subject of the article.
• Category B: OPG forms a defined and identifiable component of a broader question, most often the tumor spectrum of NF1, the management of pediatric low-grade glioma, or the late effects of treatment directed at these tumors.
• Category C: The record contains no substantive OPG content and was retrieved through co-occurrence of visual-pathway and glioma index terms, typically where the optic radiation features as adjacent surgical anatomy or where a general glioma model or cohort is described.

The category assigned to every record is reported in the final column of Table 1, so that readers can inspect and, if they wish, contest each judgment. All 100 records were retained in the primary analysis in order to preserve the integrity of a citation-ranked frame and to avoid the selective removal of records after their citation counts were known. The influence of the category C records was then quantified directly in a prespecified sensitivity analysis (Section 2.9).

**Table 1:** The 100 most-cited publications on optic nerve and optic pathway glioma, ranked by total citations, with the relevance category assigned during eligibility assessment.

Rank	Title	Year	Total citations	Citations per year	Relevance category
1	Proton versus photon radiotherapy for common pediatric brain tumors: comparison of models of dose characteristics and their relationship to cognitive function	2008	289	17.00	B
2	Moyamoya following cranial irradiation for primary brain tumors in children	2007	281	15.61	B
3	Visual outcomes in children with neurofibromatosis type 1-associated optic pathway glioma following chemotherapy: a multicenter retrospective analysis	2012	263	20.23	A
4	Spontaneous regression of optic gliomas: 13 cases documented by serial neuroimaging	2001	256	10.66	A
5	Proteomic analysis reveals hyperactivation of the mammalian target of rapamycin pathway in neurofibromatosis-1-associated human and mouse brain tumors	2005	255	12.75	B
6	Phase II weekly vinblastine for chemotherapy-naïve children with progressive low-grade glioma: a Canadian Pediatric Brain Tumor Consortium study	2016	193	21.44	B
7	Neurofibromatosis type 1 and optic pathway gliomas: follow-up of 54 patients	2004	190	9.04	A
8	Visual loss in children with neurofibromatosis type 1 and optic pathway gliomas: relation to tumor location by magnetic resonance imaging	2001	170	7.08	A
9	Optic pathway glioma: correlation of imaging findings with the presence of neurofibromatosis	2001	163	6.79	A
10	Inactivation of NF1 in CNS causes increased glial progenitor proliferation and optic glioma formation	2005	162	8.10	A
11	Optic pathway gliomas in children with and without neurofibromatosis-1	2003	146	6.63	A
12	Natural history and outcome of optic pathway gliomas in children	2009	143	8.93	A
13	Implementation of fiber tract navigation	2006	140	7.36	C
14	Cancer and central nervous system tumor surveillance in pediatric neurofibromatosis-1	2017	139	17.37	B
15	Optic pathway gliomas in neurofibromatosis type 1	2018	134	19.14	A
16	NF1 mutation drives neuronal activity-dependent initiation of optic glioma	2021	130	32.50	A
17	Factors influencing neurocognitive outcomes in young patients with benign and low-grade brain tumors treated with stereotactic conformal radiotherapy	2010	125	8.33	B
18	Long-term outcome in children with gliomas of the anterior visual pathway	2003	124	5.63	A
19	Temozolomide in children with progressive low-grade glioma	2007	120	6.66	B
20	Natural history and clinical management of optic pathway glioma	2003	119	5.40	A
21	Sex is a major determinant of neuronal dysfunction in neurofibromatosis type 1	2014	118	10.72	B
22	Late-onset optic pathway tumors in children with neurofibromatosis-1	2004	116	5.52	A
23	A phase II trial of selumetinib in children with recurrent optic pathway and hypothalamic low-grade glioma without NF1: a Pediatric Brain Tumor Consortium study	2021	110	27.50	A
24	Radiation therapy of optico-hypothalamic gliomas: radiographic response, vision and late toxicity	2000	109	4.36	A
25	Functional outcome measures for NF1-associated optic pathway glioma clinical trials	2013	109	9.08	A
26	Radiological classification of optic pathway gliomas: experience of a modified functional classification system	2008	108	6.35	A
27	Carboplatin is effective therapy for young children with progressive optic pathway tumors: a Pediatric Oncology Group phase II study	2000	105	4.20	A
28	Clinical spectrum of individuals with pathogenic NF1 missense variants affecting p.Met1149, p.Arg1276, and p.Lys1423: genotype–phenotype study in neurofibromatosis type 1	2020	105	21.00	B
29	Single- and multi-fraction stereotactic radiosurgery dose tolerances of the optic pathways	2021	103	25.75	B
30	New insights into the anatomic dissection of the temporal stem with special emphasis on the inferior fronto-occipital fasciculus: implications in surgical approach to left mesiotemporal and temporoinsular structures	2010	103	6.86	C
31	Marked recovery of vision in children with optic pathway gliomas treated with bevacizumab	2014	102	9.27	A
32	Glioma formation in neurofibromatosis-1 reflects preferential activation of K-RAS in astrocytes	2005	102	5.10	B
33	Optic pathway hypothalamic gliomas in children under 3 years of age: the role of chemotherapy	2000	99	3.96	A
34	Cyclic AMP suppression is sufficient to induce gliomagenesis in a mouse model of neurofibromatosis-1	2010	98	6.53	B
35	Defective cAMP generation underlies the sensitivity of CNS neurons to neurofibromatosis-1 heterozygosity	2010	98	6.53	B
36	Spatiotemporal differences in CXCL12 expression and cyclic AMP underlie the unique pattern of optic glioma growth in neurofibromatosis type 1	2007	97	5.38	A
37	Low doses of PEG-coated gold nanoparticles sensitize solid tumors to cold plasma by blocking the PI3K/AKT-driven signaling axis to suppress cellular transformation by inhibiting growth and EMT	2016	96	10.66	C
38	Neoplasms associated with germline and somatic NF1 gene mutations	2012	94	7.23	B
39	Increased c-Jun-NH2-kinase signaling in neurofibromatosis-1 heterozygous microglia drives microglia activation and promotes optic glioma proliferation	2008	93	5.47	A
40	Visual outcome of a cohort of children with neurofibromatosis type 1 and optic pathway glioma followed by a pediatric neuro-oncology program	2007	92	5.11	A
41	Retinal nerve fiber layer thickness in children with optic pathway gliomas	2011	90	6.42	A
42	Neuroendocrine morbidity after pediatric optic gliomas: a longitudinal analysis of 166 children over 30 years	2015	88	8.80	A
43	The use of magnetic resonance imaging screening for optic pathway gliomas in children with neurofibromatosis type 1	2015	85	8.50	A
44	Proceedings from the 2009 genetic syndromes of the Ras/MAPK pathway: from bedside to bench and back	2010	83	5.53	B
45	A molecular analysis of individuals with neurofibromatosis type 1 (NF1) and optic pathway gliomas (OPGs), and an assessment of genotype–phenotype correlations	2011	82	5.85	A
46	Akt- or MEK-mediated mTOR inhibition suppresses Nf1 optic glioma growth	2015	81	8.10	A
47	Fractionated stereotactic radiotherapy of optic pathway gliomas: tolerance and long-term outcome	2005	79	3.95	A
48	Neurofibromatosis-1 regulates mTOR-mediated astrocyte growth and glioma formation in a TSC/Rheb-independent manner	2011	78	5.57	B
49	MRI and clinical differences between optic pathway tumors in children with and without neurofibromatosis	2001	76	3.16	A
50	Hypothalamic-pituitary lesions in pediatric patients: endocrine symptoms often precede neuro-ophthalmic presenting symptoms	2012	75	5.76	B
51	Pediatric optical coherence tomography in clinical practice: recent progress	2016	75	8.33	B
52	Optic pathway glioma: long-term visual outcome in children without neurofibromatosis type 1	2010	74	4.93	A
53	Phase I dose-escalation study of the PI3K/mTOR inhibitor voxtalisib (SAR245409, XL765) plus temozolomide with or without radiotherapy in patients with high-grade glioma	2015	71	7.10	C
54	Optic gliomas: a retrospective analysis of 50 cases	2003	71	3.22	A
55	Ganglion cell layer-inner plexiform layer thickness and vision loss in young children with optic pathway gliomas	2014	70	6.36	A
56	Optic pathway gliomas in patients with neurofibromatosis type 1: follow-up of 44 patients	2010	70	4.66	A
57	Automatic segmentation, internal classification, and follow-up of optic pathway gliomas in MRI	2012	68	5.23	A
58	Histopathologic predictors of pilocytic astrocytoma event-free survival	2009	68	4.25	B
59	Spectrum and prevalence of vasculopathy in pediatric neurofibromatosis type 1	2013	66	5.50	B
60	Optical coherence tomography in the evaluation of neurofibromatosis type 1 subjects with optic pathway gliomas	2010	66	4.40	A
61	Natural history of neurofibromatosis-1-associated optic nerve glioma in mice	2005	66	3.30	A
62	Visual outcome following chemotherapy for progressive optic pathway gliomas	2011	65	4.64	A
63	Activation of mTORC1/mTORC2 signaling in pediatric low-grade glioma and pilocytic astrocytoma reveals mTOR as a therapeutic target	2013	65	5.41	B
64	Systematic MRI in NF1 children under 6 years of age for the diagnosis of optic pathway gliomas: study and outcome of a French cohort	2016	65	7.22	A
65	Can morphological MRI differentiate between primary central nervous system lymphoma and glioblastoma?	2016	64	7.11	C
66	Modeling glioma growth and invasion in *Drosophila melanogaster*	2009	63	3.93	C
67	Neurofibromatosis type 1 and high-grade tumors of the central nervous system	2010	63	4.20	B
68	BRAF duplications and MAPK pathway activation are frequent in gliomas of the optic nerve proper	2012	62	4.76	A
69	Radiation therapy for optic pathway and hypothalamic low-grade gliomas in children	2017	62	7.75	A
70	Longitudinal change of circumpapillary retinal nerve fiber layer thickness in children with optic pathway gliomas	2015	60	6.00	A
71	Growth and pubertal disorders in neurofibromatosis type 1	2003	60	2.72	B
72	Ophthalmological manifestations in segmental neurofibromatosis type 1	2004	60	2.85	B
73	Neuropsychological outcome in children with optic pathway tumors when 1st-line treatment is chemotherapy	2003	60	2.72	A
74	Long-term visual outcome after chemotherapy for optic pathway glioma in children: site and age are strongly predictive	2015	59	5.90	A
75	Estrogen activation of microglia underlies the sexually dimorphic differences in NF1 optic glioma-induced retinal pathology	2017	59	7.37	A
76	Nerve growth factor improves visual loss in childhood optic gliomas: a randomized, double-blind, phase II clinical trial	2016	58	6.44	A
77	The course and the anatomo-functional relationships of the optic radiation: a combined study with post mortem dissections and in vivo direct electrical mapping	2015	58	5.80	C
78	Progression from 1st symptom to diagnosis in childhood brain tumors	2012	58	4.46	B
79	Visual outcomes after chemotherapy for optic pathway glioma in children with and without neurofibromatosis type 1: results of the International Society of Paediatric Oncology (SIOP) Low-Grade Glioma 2004 trial UK cohort	2018	57	8.14	A
80	Long-term visual outcomes of optic pathway gliomas in pediatric patients without neurofibromatosis type 1	2016	57	6.33	A
81	Pilocytic astrocytoma of the optic pathway: a tumor deriving from radial glia cells with a specific gene signature	2009	56	3.50	A
82	Deep learning guided partitioned shape model for anterior visual pathway segmentation	2016	56	6.22	B
83	Optic radiation involvement in optic pathway gliomas in neurofibromatosis	2004	56	2.66	A
84	Intraoperative high-field magnetic resonance imaging combined with fiber tract neuronavigation-guided resection of cerebral lesions involving optic radiation	2011	55	3.92	C
85	Nonperioperative strokes in children with central nervous system tumors	2002	54	2.34	B
86	Vascularity and angiogenesis as predictors of growth in optic pathway/hypothalamic gliomas	2006	53	2.78	A
87	Handheld optical coherence tomography during sedation in young children with optic pathway gliomas	2014	53	4.81	A
88	Ascites following ventriculoperitoneal shunting in children with chiasmatic-hypothalamic glioma	2001	51	2.12	A
89	Longitudinal measures of visual function, tumor volume, and prediction of visual outcomes after treatment of optic pathway gliomas	2012	49	3.76	A
90	Topical nerve growth factor as a visual rescue strategy in pediatric optic gliomas: a pilot study including electrophysiology	2011	48	3.42	A
91	Optic nerve dysfunction in a mouse model of neurofibromatosis-1 optic glioma	2009	47	2.93	A
92	Does attention-deficit-hyperactivity disorder exacerbate executive dysfunction in children with neurofibromatosis type 1?	2012	46	3.53	C
93	Can screening for optic nerve gliomas in patients with neurofibromatosis type 1 be performed with visual-evoked potential testing?	2006	46	2.42	A
94	A fast visual-evoked potential method for functional assessment and follow-up of childhood optic gliomas	2004	45	2.14	A
95	Clinical presentation and outcome of patients with optic pathway glioma	2017	44	5.50	A
96	Natural history of optic pathway gliomas in a cohort of unselected patients affected by neurofibromatosis-1	2017	43	5.37	A
97	Single agent vinorelbine in pediatric patients with progressive optic pathway glioma	2015	42	4.20	A
98	Fractional anisotropy of the optic radiations is associated with visual acuity loss in optic pathway gliomas of neurofibromatosis type 1	2013	42	3.50	A
99	Endocrine long-term follow-up of children with neurofibromatosis type 1 and optic pathway glioma	2017	42	5.25	A
100	Epilepsy in neurofibromatosis type 1	2017	41	5.12	B

Records were identified in Scopus on April 30, 2025 and are ranked by total citations at that date. Citations per year is the total citation count divided by the number of complete years since publication.

Relevance categories were assigned independently by 2 reviewers, with a 3rd resolving disagreement. Category A: optic pathway or optic nerve glioma is the primary subject of the article (n = 63). Category B: optic pathway glioma forms a defined and identifiable component of a broader question, most often the tumor spectrum of neurofibromatosis type 1, the management of pediatric low-grade glioma, or the late effects of treatment directed at these tumors (n = 28). Category C: the record contains no substantive optic pathway glioma content and was retrieved through co-occurrence of visual-pathway and glioma index terms (n = 9); these rows are shaded. All 100 records were retained in the primary analysis, and the Category C records were removed in a prespecified sensitivity analysis reported in Section 3.4 of the manuscript.

AMP = adenosine monophosphate, cAMP = cyclic adenosine monophosphate, CNS = central nervous system, EMT = epithelial–mesenchymal transition, MAPK = mitogen-activated protein kinase, MEK = mitogen-activated protein kinase kinase, MRI = magnetic resonance imaging, mTOR = mechanistic target of rapamycin, mTORC = mechanistic target of rapamycin complex, NF1 = neurofibromatosis type 1, OPG = optic pathway glioma, PEG = polyethylene glycol, PI3K = phosphoinositide 3-kinase, SIOP = International Society of Paediatric Oncology, TSC = tuberous sclerosis complex, UK = United Kingdom.

### 2.6. Study selection

Records retrieved by the search were exported and sorted in descending order of total citations, and the 100 most-cited were taken forward as the citation-ranked sampling frame. Titles and abstracts were screened independently by 2 reviewers (MHS and SaR), with disagreements resolved by discussion with a 3rd reviewer (SuR). Full records were then retrieved and assessed against the eligibility criteria and the relevance categories described above. The selection process, with the number of records at each stage, is shown in Figure 1.

**Figure 1.**
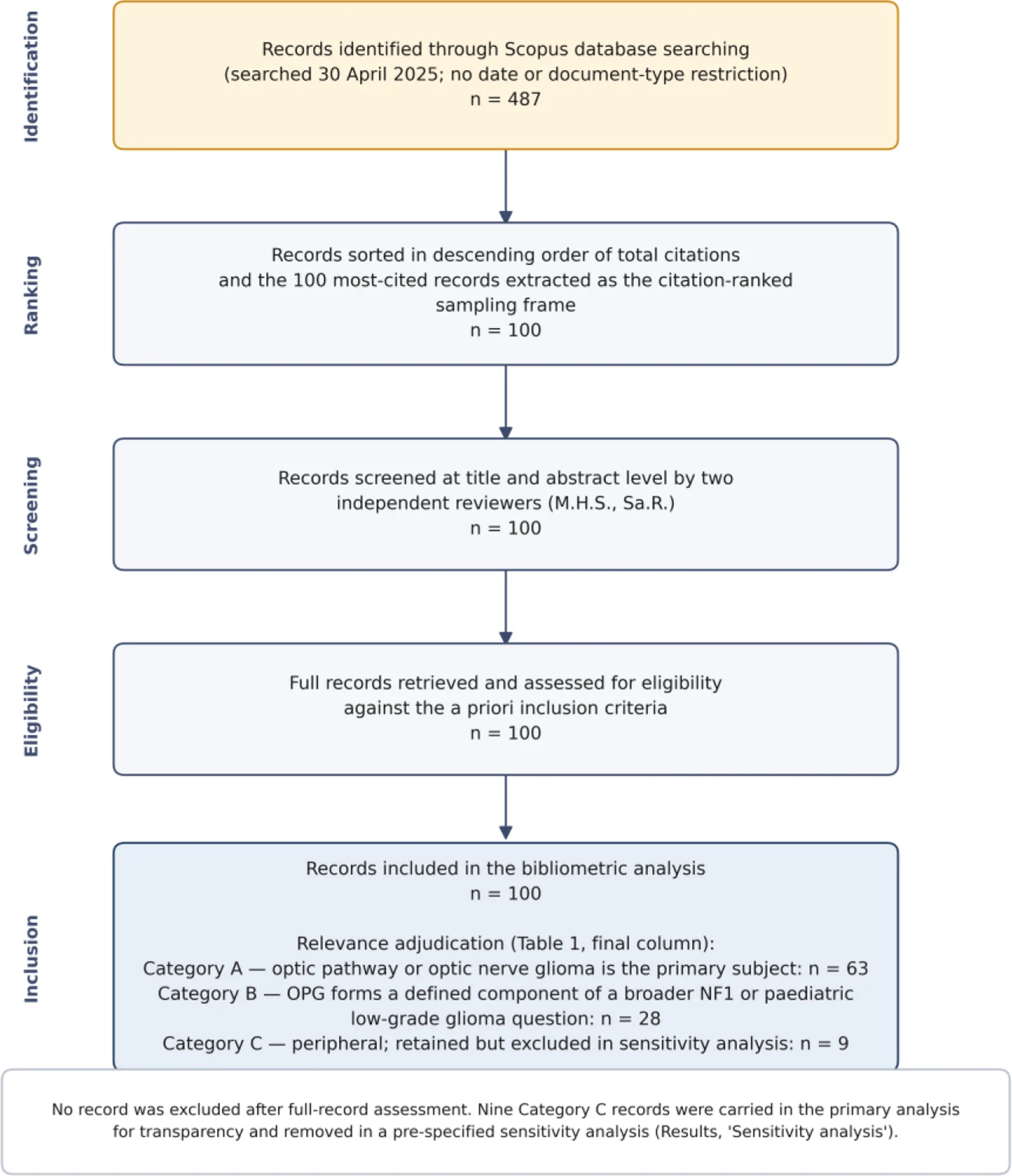
Selection of the 100 most-cited publications on optic nerve and optic pathway glioma. Records were identified in Scopus on April 30, 2025, ranked by total citations, and the 100 most-cited were carried forward as the citation-ranked sampling frame. Screening and eligibility assessment were performed independently by 2 reviewers with a 3rd resolving disagreement. The final panel shows the relevance category assigned to each included record: Category A, optic pathway or optic nerve glioma is the primary subject; Category B, OPG forms a defined component of a broader question; Category C, no substantive OPG content. Category C records were retained in the primary analysis and removed in a prespecified sensitivity analysis. NF1 = neurofibromatosis type 1, OPG = optic pathway glioma.

### 2.7. Data extraction and standardization

Eligible records were exported in comma-separated and BibTeX formats. The following fields were extracted: title, full author list, publication year, document type, source journal, total citations, annual citation rate, all author affiliations, country of the 1st author’s affiliation, author and index keywords, and funding information where recorded.

Author disambiguation was carried out using the Scopus Author Identifier as the primary key rather than the name string, since the identifier is stable across changes in the form of a name. Records sharing an identifier were merged. Where an identifier was absent, name variants were reconciled manually by comparing affiliation history, coauthor sets, and subject area; this affected differences in the use of middle initials, hyphenation, and diacritics. Keyword variants were consolidated manually before analysis, so that singular and plural forms and recognized synonyms (for example, “optic nerve glioma” and “optic glioma”) were treated as a single term.

Institutional productivity was derived from the affiliation field of every author on a record, not from 1st-author or corresponding-author affiliation alone. Two properties of this counting method should be borne in mind when reading Figure 3. Bibliometrix tabulates each author’s affiliation entry separately, so a publication carrying several authors from the same institution contributes >1 count; the values in Figure 3 are therefore affiliation instances rather than distinct publications, and they may exceed the number of articles from the corresponding country. In addition, Bibliometrix treats affiliation strings literally, so departmental or school-level variants of the same university are tabulated as separate entities. Both “Washington University School of Medicine” and “Washington University” consequently appear in Figure 3, and the 2 entries refer to the same institution. We have retained the unconsolidated output rather than merging the strings, because the number of distinct publications underlying the merged total cannot be recovered from the affiliation-instance counts without reidentifying every author record, and a merged figure would give a false impression of precision. This is stated explicitly in the legend to Figure 3 and in the Section 4.2.

**Figure 2.**
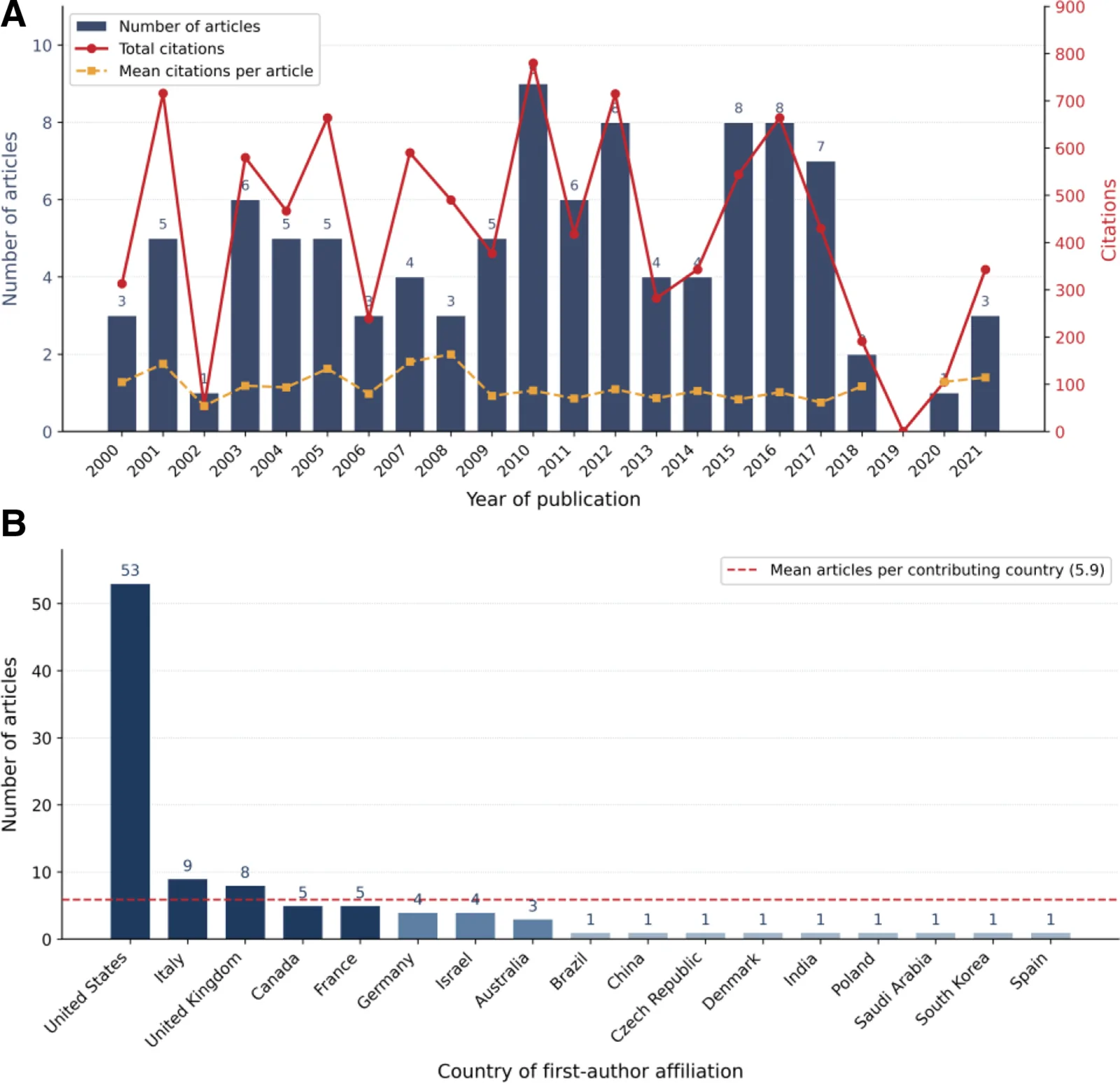
(A) Annual distribution of the 100 most-cited publications on optic pathway glioma, 2000 to 2021. Bars show the number of articles published in each year (left axis). The solid line shows the total citations accrued by the articles published in that year and the dashed line the mean citations per article for that year (right axis). Values above each bar give the number of articles. (B) Distribution of the 100 most-cited publications on optic pathway glioma by country of 1st-author affiliation. Values above each bar give the number of articles. The dashed line marks the mean number of articles per contributing country (5.9 across 17 countries).

### 2.8. Bibliometric and visualization tools

Three tools were used. Biblioshiny, the web interface of the Bibliometrix package for R (Aria M, Cuccurullo C; K-Synth Srl), was used for descriptive analysis, affiliation ranking, author production over time, and the country collaboration map. VOSviewer version 1.6.18 (Centre for Science and Technology Studies, Leiden University) was used for co-authorship and keyword co-occurrence mapping. Microsoft Excel was used for data cleaning and for tabulation. In the network figures, each node represents an entity such as an author or a keyword, the links between nodes indicate collaboration or co-occurrence, line thickness reflects the strength of the relationship, and node size reflects publication frequency or citation volume.

### 2.9. Journal metrics

Impact factors for source journals were taken from the 2023 Journal Citation Reports (Clarivate Analytics), the most recent edition available at the time of analysis. Journals were also assigned to Science Citation Index Expanded quartiles in order to describe the distribution of publication venues. Impact factors are reported as descriptive information about the journals in which this literature appeared; they are not used as a measure of the quality of individual articles.

### 2.10. Statistical analysis

Normality was assessed using the Shapiro–Wilk test, which is appropriate for samples of fewer than 200 observations. Total citations (*W* = 0.780) and citations per year (*W* = 0.703) both departed markedly from normality (*P* < .001 for each), so continuous variables are reported as medians with interquartile ranges (IQRs) and ranges rather than as means with standard deviations. Associations between continuous variables were examined using Spearman rank correlation coefficient (ρ). Citation rates between publication periods were compared using the Mann–Whitney *U* test. Two-sided *P* values below .05 were considered statistically significant.

A sensitivity analysis was specified before the results were interpreted, in which all citation-level analyses were repeated after excluding the category C records, and again after restricting the dataset to category A records alone. This was done to establish whether the composition of the retrieved set had any material bearing on the descriptive findings.

Analyses and the statistical figures were produced in Python version 3.11 (Python Software Foundation) using the pandas, SciPy, and Matplotlib libraries. The correlation between total citations and citations per year is reported for completeness but is not interpreted as a substantive finding, for the reasons set out in Section 3.3.

## 3. Results

### 3.1. Characteristics of the most-cited articles

One hundred articles were included (Table 1). Together, they had been cited 9305 times at the date of extraction. The median number of citations per article was 75 (IQR 59–108; range 41–289) and the median annual citation rate was 5.7 (IQR 4.2–7.8). The distribution was strongly right-skewed: a small group of articles accounted for a disproportionate share of the total, with the 10 most-cited accruing 2222 citations, or approximately a quarter of all citations in the set.

The most-cited article was a 2008 modeling study comparing proton with photon radiotherapy for common pediatric brain tumors, with 289 citations (17.0/yr). The highest annual citation rate belonged to a 2021 laboratory study showing that NF1 mutation drives neuronal activity-dependent initiation of optic glioma, which had accumulated 130 citations at 32.5/yr. We note explicitly that the mean number of citations per article in this set is 93.1; the value of 101.6 reported in the previous version of this manuscript was an arithmetic error, and the corrected figures are used throughout.

### 3.2. Temporal distribution and citation velocity

The included articles were published between 2000 and 2021 (Fig. 2A). Output was highest in 2010 (n = 9), followed by 2012, 2015, and 2016 (n = 8 each), and 2017 (n = 7). Only 1 article published in 2002 and 1 published in 2020 entered the set, and no article published in 2019 did so. Numbers fall away after 2017, which is most plausibly explained by citation lag rather than by any decline in research activity: recent work has had less time to accumulate the citations needed to enter a citation-ranked set.

Publication year correlated positively and moderately with annual citation rate (ρ = 0.475, *P* < .001), indicating that among these highly cited articles, the more recent ones are being cited at a faster rate. The same pattern is evident when the set is divided at 2010: articles published in 2011 or later had a median annual citation rate of 6.4, compared with 5.1 for articles published in 2010 or earlier (Mann–Whitney *U* test, *P* < .001). Publication year correlated negatively with total citations (ρ = −0.245, *P* = .014), as expected, since older articles have had longer to accumulate them.

### 3.3. A note on the relationship between total citations and citation rate

Total citations and citations per year were strongly correlated (ρ = 0.680, *P* < .001). We report this for completeness but do not treat it as a substantive result. Citations per year is derived by dividing total citations by the number of years since publication, so the 2 measures are arithmetically linked, and a positive association between them is expected in any citation dataset. It carries no information about the field beyond the fact that the derivation behaved as it should. The association that does carry information is the 1 between publication year and citation rate, reported in Section 3.2. The corresponding statement in the previous version of this manuscript, which presented the total-citation to citation-rate correlation as evidence that highly cited articles also attract citations faster, has been removed.

### 3.4. Relevance adjudication and sensitivity analysis

Of the 100 records, 63 were classified as category A, in which OPG was the primary subject; 28 as category B, in which OPG formed an identifiable component of a broader question, most often the tumor spectrum of NF1, the management of pediatric low-grade glioma, or the late effects of treatment; and 9 as category C, in which no substantive OPG content was present. The category C records were retrieved through co-occurrence of visual-pathway and glioma index terms and fall into 2 recognizable groups. Four concern the optic radiation as adjacent white-matter anatomy in tumor surgery or in anatomical dissection studies (ranks 13, 30, 77, and 84 in Table 1). The remaining 5 are general neuro-oncological or NF1 studies in which the anterior visual pathway is not examined (ranks 37, 53, 65, 66, and 92). The category assigned to each of the 100 records is given in Table 1.

Removing the 9 category C records left the descriptive picture unchanged. Across the remaining 91 articles, the median was 76 citations (IQR 59–109) against 75 (IQR 59–108) in the full set; the median annual citation rate was 5.6 against 5.7, and the correlation between publication year and citation rate was ρ = 0.483 against ρ = 0.475. Restricting the analysis further to the 63 category A articles gave a median of 71 citations (IQR 57–109), a median annual rate of 5.5, and ρ = 0.455 (*P* < .001) between publication year and citation rate. None of the conclusions drawn in this article depend on whether the category B or category C records are included.

### 3.5. Country of 1st-author affiliation

First authors were affiliated with institutions in 17 countries (Fig. 2B). The United States accounted for 53 of the 100 articles, more than the remaining 16 countries combined. Italy followed with 9 articles and the United Kingdom with 8, then France and Canada with 5 each, Germany and Israel with 4 each, and Australia with 3. Nine countries contributed a single article: Brazil, China, the Czech Republic, Denmark, India, Poland, Saudi Arabia, South Korea, and Spain. No 1st author was affiliated with an institution in Africa, and none in Latin America apart from the single Brazilian record. The corrected counts for the United States and the United Kingdom differ by 1 article from those reported in the previous version of this manuscript, in which they were misstated as 54 and 7 respectively.

### 3.6. Institutional contributions

Washington University School of Medicine occurred most frequently in the affiliation field, with 56 affiliation instances, followed by Duke University Medical Center and the University of Pennsylvania School of Medicine with 20 each (Fig. 3). As set out in Section 2.7, these are counts of author affiliation entries rather than of distinct publications, which is why the leading value exceeds the number of articles with a United States 1st author; a paper carrying several authors from 1 institution contributes >1 instance. “Washington University” also appears separately in Figure 3 and refers to the same institution as “Washington University School of Medicine.” These figures should therefore be read as an indication of the relative concentration of authorship rather than as exact publication counts.

### 3.7. Authorship and collaboration

David H. Gutmann and Robert A. Avery were the most prominent authors in the set. Gutmann published steadily and with sustained citation impact between 2001 and 2020, while Avery contribution was concentrated between 2004 and 2013 (Fig. 4A). Other authors contributed more sporadically and with lower citation density. The co-authorship network placed Gutmann at the center of the largest cluster, with close links to Joshua Rubin and Jacquelyn Brown; separate clusters formed around Avery and colleagues and around groups working on chemotherapy outcomes (Fig. 4B). International collaboration was centered on North America, with links to Europe, Israel, and Australia (Fig. 4C).

The number of authors on an article showed no meaningful association with its total citations (ρ = 0.081, *P* = .425). Within this set, therefore, team size does not appear to have influenced citation accumulation.

### 3.8. Journals

The 100 articles appeared in 39 journals (Table 2). Neuro-Oncology published the largest number (n = 9; 2023 impact factor 16.4), followed by Cancer Research (n = 5; 12.5) and the International Journal of Radiation Oncology Biology Physics (n = 5; 6.1). Approximately four-fifths of the articles were published in 1st-quartile journals. The spread across oncology, radiation oncology, ophthalmology, neurology and pediatric journals reflects the position of OPG at the intersection of several specialties rather than any single disciplinary home.

**Table 2:** Leading journals in the dissemination of high-impact optic pathway gliomas (OPG) research.

Rank	Journal	Articles	Total citations	Impact factor (2023)	JCR quartile
1	Neuro‑Oncology	9	949	16.4	*Q*1
2	Cancer Research	5	645	12.5	*Q*1
3	International Journal of Radiation Oncology Biology Physics	5	440	6.1	*Q*1
4	American Journal of Ophthalmology	4	376	4.1	*Q*1
5	Pediatric Blood & Cancer	4	571	2.9	*Q*1
6	Journal of Neuro‑Oncology	3	142	3.2	*Q*2
7	Journal of AAPOS	3	182	1.2	*Q*2
8	Neurology	3	506	8.3	*Q*1
9	Neurosurgery	3	298	4.1	*Q*1
10	Journal of Child Neurology	3	109	2.0	*Q*2

AAPOS = American Association for Pediatric Ophthalmology and Strabismus, JCR = Journal Citation Reports.

### 3.9. Keyword co-occurrence and thematic composition

Keyword mapping identified “optic nerve glioma,” “neurofibromatosis type 1,” “visual acuity” and “tumor growth” as the most frequent terms (Fig. 5). Three clusters were evident. The 1st brought together clinical outcome and chemotherapy terms, including vinblastine and carboplatin. The 2nd grouped molecular and genetic terms, including neurofibromin, signal transduction and animal models. The 3rd contained imaging and diagnostic terms, including optical coherence tomography, diffusion tensor imaging and visual fields. Chemotherapy terms were concentrated in the older literature, while molecular and imaging terms occurred more often in recent work.

**Figure 3.**
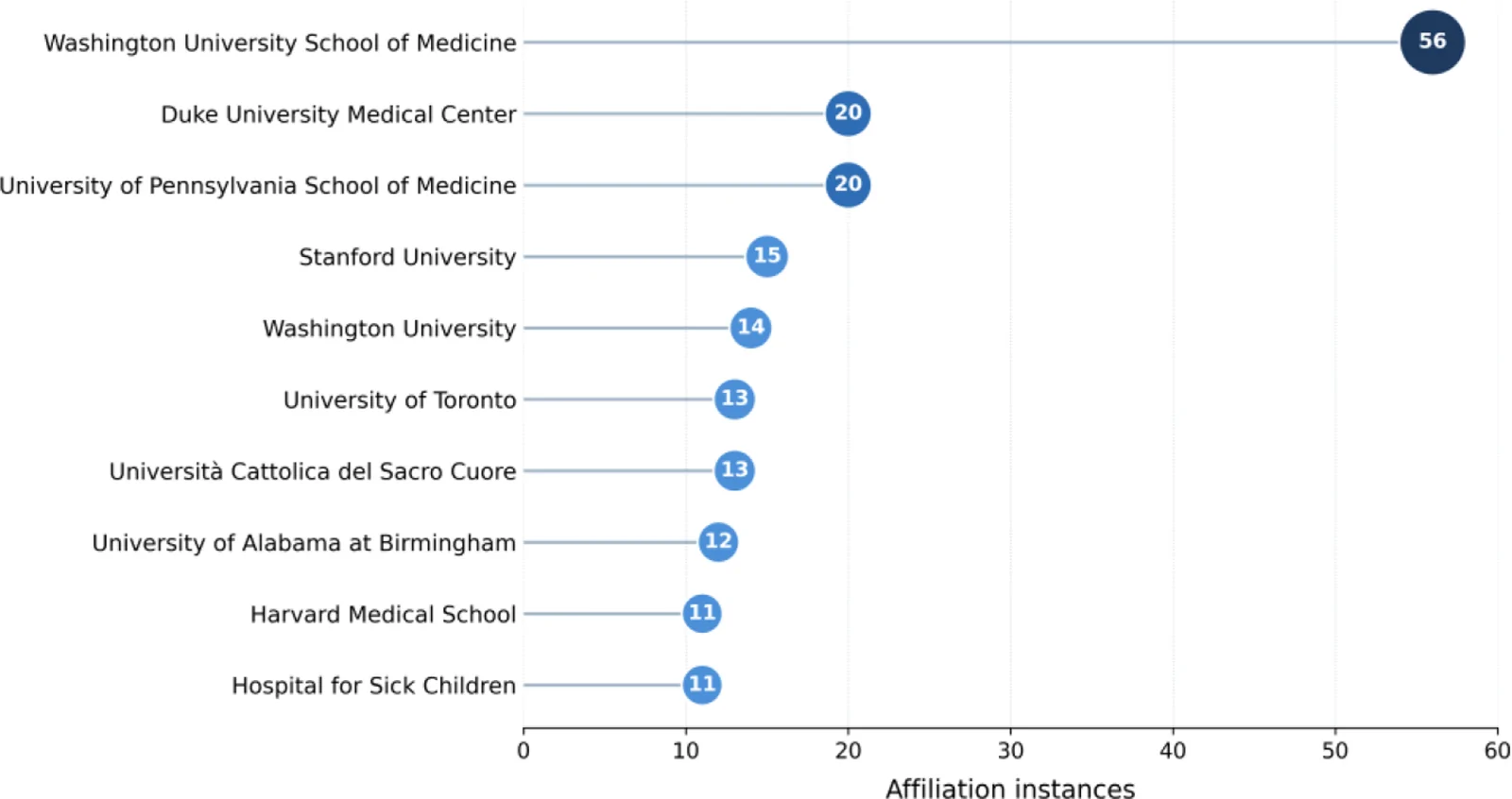
Institutions occurring most frequently in the affiliation field of the 100 most-cited publications on optic pathway glioma. Values are affiliation instances derived from all author affiliations, not counts of distinct publications: a publication carrying several authors from 1 institution contributes >1 instance, so the totals may exceed the number of articles from the corresponding country. Bibliometrix treats affiliation strings literally and does not merge name variants; “Washington University School of Medicine” and “Washington University” therefore appear as separate entries and refer to the same institution.

**Figure 4.**
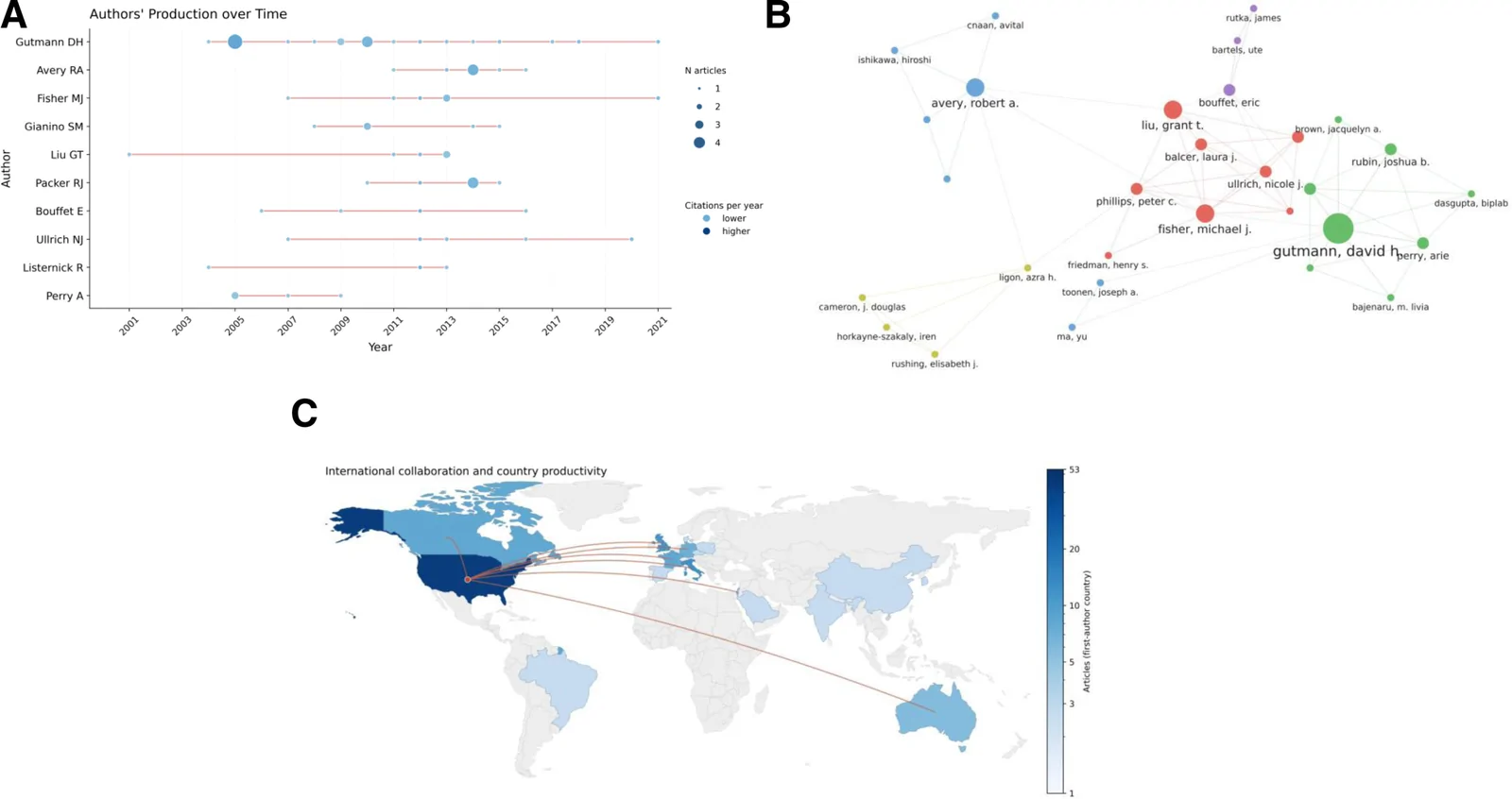
(A) Publication activity over time for the most productive authors in the 100 most-cited publications on optic pathway glioma. Circle size indicates the number of articles published in that year, and color intensity indicates the total citations per year. (B) Co-authorship network of authors contributing to the 100 most-cited publications on optic pathway glioma. Node size reflects the number of publications, node color indicates cluster membership, and links indicate co-authorship, with line thickness proportional to the strength of the association. (C) International collaboration in the 100 most-cited publications on optic pathway glioma. Shading indicates the number of publications per country, and the connecting lines indicate collaborative links between countries.

**Figure 5.**
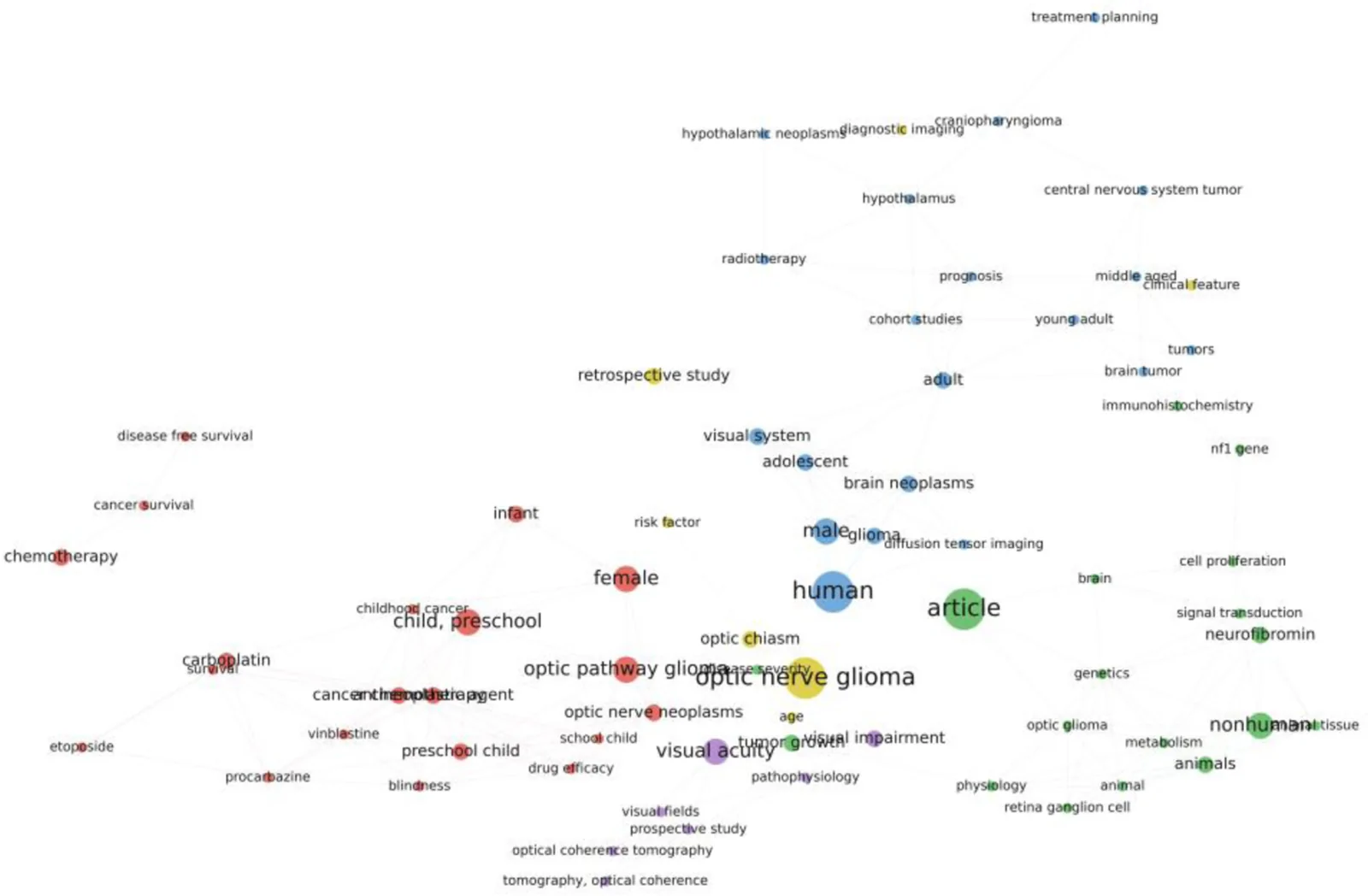
Keyword co-occurrence map for the 100 most-cited publications on optic pathway glioma. Node size reflects the frequency of the term, color indicates cluster membership, and proximity and link thickness reflect the frequency of co-occurrence.

### 3.10. Most-cited review articles

The 10 most-cited reviews were published between 2006 and 2019 (Table 3). The most-cited was “Optic pathway gliomas: a review,” published in Neurosurgical Focus in 2007, with 161 citations. The highest annual citation rate belonged to “Pediatric low-grade gliomas: next biologically driven steps,” published in Neuro-Oncology in 2018, at 19.0 citations/yr. Seven of the 10 were published more than a decade before the search date, which suggests that older synthetic work continues to be drawn on. Only 3 appeared after 2015, although these more recent reviews show higher annual citation rates, consistent with the pattern described in Section 3.2. The European Journal of Cancer contributed 2 of the 10.

**Table 3:** The 10 most cited review articles on optic pathway glioma (OPG).

Rank	Name	Year	Journal	Cited by	Citations per year
1	Optic pathway gliomas: a review	2007	Neurosurgical Focus	161	8.94
2	Prognostic factors for progression of childhood optic pathway glioma: a systematic review	2006	European Journal of Cancer	138	7.26
3	Pediatric low-grade gliomas: next biologically driven steps	2018	Neuro-Oncology	133	19.0
4	Radiation-induced moyamoya syndrome	2006	International Journal of Radiation Oncology Biology Physics	118	6.21
5	Does chemotherapy affect the visual outcome in children with optic pathway glioma? A systematic review of the evidence	2010	European Journal of Cancer	89	5.93
6	Review and update of SPRED1 mutations causing legius syndrome	2012	Human Mutation	88	6.76
7	Neurofibromatosis type 1: review and update on emerging therapies	2019	Asia-Pacific Journal of Ophthalmology	48	8.00
8	Utility of positron emission tomography for tumor surveillance in children with neurofibromatosis type 1	2010	European Journal of Nuclear Medicine and Molecular Imaging	40	2.66
9	Optic pathway gliomas: neoplasms, not hamartomas	2013	JAMA Ophthalmology	22	1.83
10	Optic pathway gliomas secondary to neurofibromatosis type 1	2017	Seminars in Pediatric Neurology	21	2.62

## 4. Discussion

This analysis describes how citations have been distributed across 2 decades of OPG research and how the composition of the most-cited literature has changed. Three observations stand out: the citation record is dominated by a small number of articles and a small number of centers, the thematic center of gravity has moved from clinical description toward tumor biology and quantitative imaging, and the most recent work in the set is being cited at a faster rate than the older work it has joined.

The concentration of citations is pronounced. The 10 most-cited articles account for roughly a quarter of all citations in the set, and the IQR of 59 to 108 citations sits well below the maximum of 289. The articles at the top of the ranking are of 2 kinds. The 1st are studies addressing questions with immediate bearing on how children are treated, such as the 2008 comparison of proton and photon radiotherapy, which spoke to a longstanding concern about cognitive late effects in pediatric brain tumor patients.^[18]^ The 2nd are early clinical observations that reframed how the natural history of these tumors was understood, notably the 2001 series documenting spontaneous regression of optic gliomas and the 2004 follow-up of 54 patients with NF1-associated disease.^[19,20]^ Alongside these sit the chemotherapy and radiotherapy studies that defined practice for much of the period covered.^[21,22]^

The more recent entries have a different character. The 2021 report that NF1 mutation drives neuronal activity-dependent initiation of optic glioma had accrued 130 citations within 4 years of publication, at >30/yr.^[23]^ Its rapid uptake is consistent with the shift visible in the keyword map, where chemotherapeutic agents such as vinblastine and carboplatin cluster with the older literature while neurofibromin, signal transduction, and animal models cluster with the newer. The same movement is evident in the pattern of citation rates: articles published from 2011 onward are cited at a higher annual rate than those published earlier, and publication year correlates positively with citation velocity across the set. We would caution against reading this as evidence that recent work is more important. Citation rates are shaped by the size of the citing community, by publication volume in adjacent fields, and by the tendency of newer work to be cited while it is still novel, and a rate measured over 4 years is a less stable estimate than 1 measured over 20.

Geographically, the field is heavily concentrated. Just over half of the articles had a 1st author based in the United States, and the affiliation counts place Washington University School of Medicine well ahead of any other institution. This concentration reflects the presence of a small number of long-established groups, and the co-authorship map shows a network organized around them rather than a set of independent centers. Such a structure has advantages, in that it supports the sustained programs of work needed to move from mechanism to trial, but it also means that the questions the field asks are shaped by a narrow group of investigators, and that regions where children with NF1 are cared for but where research infrastructure is limited are largely absent from the highly cited literature. No 1st author from an African institution appears in this set. Team size, by contrast, showed no association with citation counts, which suggests that within this literature influence has followed the substance of the question rather than the number of contributors.

### 4.1. Therapeutic developments reflected in the highly cited literature

The most consequential therapeutic development represented in this set is the use of mitogen-activated protein kinase kinase (MEK) inhibitors in NF1-associated low-grade glioma.^[24]^ Phase II data for selumetinib have shown sustained responses and prolonged progression-free survival in children with recurrent or progressive disease,^[25]^ and selumetinib was approved by the United States Food and Drug Administration in 2020 for symptomatic, inoperable plexiform neurofibroma in NF1. An ongoing phase III trial comparing selumetinib with carboplatin and vincristine uses visual acuity and radiographic response as endpoints in optic pathway disease.^[26]^ Taken together with the laboratory work on Ras/mitogen-activated protein kinase signaling that appears throughout the molecular cluster, these studies mark a movement toward treatment selected on the basis of tumor biology rather than on histology and location alone. It should be said that this movement is visible in the citation record before it is established in practice: highly cited early-phase work is not the same thing as a change in standard of care, and the phase III evidence is not yet available.

Several gaps are apparent from the composition of the set. Imaging biomarkers are well represented, with optical coherence tomography measures of the retinal nerve fiber layer and the ganglion cell-inner plexiform layer appearing repeatedly, but the segmentation algorithms underlying these measures would benefit from the kind of standardization that has been pursued in glaucoma research.^[27]^ Long-term visual and endocrine outcomes after MEK inhibition have begun to be reported but remain thinly represented relative to the attention given to initial response.^[28]^ Sporadic, non-NF1 OPG is conspicuously underrepresented: most of the molecular work in this set concerns NF1-driven tumorigenesis, and the mechanisms operating in sporadic disease, which may involve non-mitogen-activated protein kinase pathways, have attracted comparatively little highly cited attention.^[29]^ These are observations about where citations have accumulated, and they should be treated as pointers to areas that may repay attention rather than as a judgment that the underlying work does not exist.

### 4.2. Limitations

Several limitations follow from the design of this study and should temper the interpretation of its findings.

First, a single database was searched. Scopus, Web of Science, PubMed, and Google Scholar differ in journal coverage, in the years for which they index citing references, and in how they attribute citations, and the citation count for a given article differs between them. A citation-ranked set assembled from Web of Science would not be identical to the 1 presented here, and both the ranking within the set and, at the margin, its membership would change. The findings should therefore be read as describing the OPG literature as represented in Scopus rather than as an absolute account of the field.

Second, the search was restricted to 4 bound phrases. Terms such as “chiasmatic glioma,” “chiasmatic-hypothalamic glioma” and “anterior visual pathway glioma” were not included as search terms. Because the title–abstract–keyword field also searches indexed keywords, most articles using these variants are retrieved through their index terms, but we cannot exclude the possibility that some relevant work was missed, particularly older articles indexed before current descriptors were standardized.

Third, retrieval through indexed keywords also works in the opposite direction, and 9 records entered the citation-ranked set without substantive OPG content. These have been identified individually in Table 1 and their influence quantified in Section 3.4, where their removal was shown not to alter any summary statistic. We have retained them in the primary analysis rather than removing them after their citation counts were known, but readers who prefer the restricted dataset will find the corresponding values reported alongside.

Fourth, only English-language articles were included. Work published in other languages, including clinical series from centers with substantial pediatric neuro-oncology practice, is excluded by design and is also systematically less likely to be cited in the English-language literature that dominates Scopus.

Fifth, citation counts are an imperfect proxy for anything a clinician or scientist might care about. They measure attention, not quality, methodological rigor, clinical importance, or current relevance. Citations accrue to review articles, to methodological papers, and to work that is contested as readily as to work that is definitive, and negative or confirmatory studies are systematically under-cited. Self-citation, citation within collaborative networks, and the tendency of authors to cite the work of prominent groups all inflate counts in ways that a bibliometric analysis cannot separate out. A highly cited article from 2001 may have been superseded, and an important recent article may not appear here at all.

Sixth, the analysis is subject to citation lag. Any citation-ranked set will underrepresent recent work, which is why only 1 article published in 2020 and none published in 2019 met the threshold for inclusion. Conclusions about the current direction of the field must be drawn cautiously from a dataset that is structurally biased toward the past.

Seventh, affiliation counts are affiliation instances rather than publication counts, and institutional name variants were not merged, for the reasons given in Section 2.7. Institutional rankings should accordingly be read as approximate. Author disambiguation relied on Scopus Author Identifiers, which are themselves generated algorithmically and occasionally split or merge author records in error.

Finally, impact factors were taken from a single edition of the Journal Citation Reports and describe journals rather than articles. They are reported here as context for where this literature appeared and carry no implication about the merit of any individual study.

## 5. Conclusion

Among the 100 most-cited publications on optic nerve and OPG indexed in Scopus, the emphasis of highly cited work has moved over 2 decades from descriptive clinical series and chemotherapy outcome studies toward NF1 tumor biology, MEK-directed therapy, and quantitative imaging. Output and citation are concentrated in a small number of North American centers and in a network organized around a few sustained research programs, and articles published from 2011 onward are being cited at a faster rate than the older work alongside which they sit.

These are descriptions of publication and citation behavior, not of clinical progress, and the distinction matters. A bibliometric analysis can show where a field has directed its attention and which questions have attracted a following; it cannot establish which findings have improved outcomes for children with these tumors, nor can it identify important work that has been overlooked. Read with that constraint in mind, the analysis suggests 3 areas in which highly cited work is thin relative to its clinical significance: standardized and validated imaging biomarkers, long-term visual and endocrine outcomes after targeted therapy, and the biology of sporadic, non-NF1 disease. It also shows literature produced by a narrow group of institutions, which is worth noting in a condition managed by clinicians worldwide. We offer this mapping as a description of the field’s structure that may be of use to investigators deciding where their effort is best directed.

## Author contributions

**Conceptualization:** Muhammad Hamza Shah, Aditya Gaur.

**Formal analysis:** Khabab Abbasher Hussien Mohamed Ahmed.

**Methodology:** Khabab Abbasher Hussien Mohamed Ahmed.

**Project administration:** Khabab Abbasher Hussien Mohamed Ahmed.

**Resources:** Khabab Abbasher Hussien Mohamed Ahmed.

**Software:** Khabab Abbasher Hussien Mohamed Ahmed.

**Supervision:** Khabab Abbasher Hussien Mohamed Ahmed.

**Validation:** Khabab Abbasher Hussien Mohamed Ahmed.

**Visualization:** Khabab Abbasher Hussien Mohamed Ahmed.

**Writing – original draft:** Muhammad Hamza Shah, Aditya Gaur, Pearl Ohenewaa Tenkorang, Fionnuala McKeown, Subham Roy, Sakshi Roy, Umar Akram, Khabab Abbasher Hussien Mohamed Ahmed.

**Writing – review & editing:** Muhammad Hamza Shah, Aditya Gaur, Pearl Ohenewaa Tenkorang, Fionnuala McKeown, Subham Roy, Sakshi Roy, Umar Akram, Khabab Abbasher Hussien Mohamed Ahmed.
